# The 2014 FDA assessment of commercial fish: practical considerations for improved dietary guidance

**DOI:** 10.1186/s12937-016-0182-9

**Published:** 2016-07-13

**Authors:** Jennifer McGuire, Jason Kaplan, John Lapolla, Rima Kleiner

**Affiliations:** 1 National Fisheries Institute, 7918 Jones Branch Dr #700, McLean, VA 22102 USA; 2 inVentiv Medical Communications, 450 West 15th Street, Suite 405, New York, NY 10011 USA

**Keywords:** Fish, Pregnancy, Diet, Mercury, Development, Cognitive

## Abstract

The U.S. Food and Drug Administration (FDA) recently released its report: *A Quantitative Assessment of the Net Effects on Fetal Neurodevelopment from Eating Commercial Fish (As Measured by IQ and also by Early Age Verbal Development in Children)*. By evaluating the benefits and potential concerns of eating fish during pregnancy and breastfeeding, the analysis suggests that pregnant women consuming two seafood meals (8–12 oz) per week could provide their child with an additional 3.3 IQ points by age 9. Recent insights from behavioral economics research indicate that other factors, such as concerns about price and methylmercury (MeHg) exposure, appear to reduce fish consumption in many individuals.

To assess the net effects of eating commercial fish during pregnancy, we compared the consumption of select fish species necessary to achieve IQ benefits with the amount necessary to have adverse developmental effects due to MeHg exposure. For the species or market types evaluated, the number of servings necessary to reach MeHg exposure to observe an adverse effect was at least twice that the amount estimated to achieve peak developmental benefit. We then reported average costs of fresh and canned or pouched fish, and calculated the cost per week for pregnant women to achieve maximum IQ benefits for their gestating child. Canned light tuna was the least expensive option at $1.83 per week to achieve maximum IQ benefit.

Due to their relatively low cost, canned and pouched fish products eaten with enough regularity are likely to provide peak cognitive benefits. Because of its popularity, canned and pouched tuna could provide some of the largest cognitive benefits from fish consumption in the U.S. Future FDA consumer advice and related educational initiatives could benefit from a broader perspective that highlights the importance of affordable and accessible fish choices. These observations underscore the importance of clear public health messaging that address both health benefits and such real-world considerations as cost and convenience.

## Introduction

In May 2014, the FDA released *A Quantitative Assessment of the Net Effects on Fetal Neurodevelopment from Eating Commercial Fish (As Measured by IQ and also by Early Age Verbal Development in Children)*. The revised 2014 report builds on current data assessing fish consumption during pregnancy and provides a scientific basis for updated dietary advice. While the 2014 report addresses a specific subset of consumers, its findings are of general interest, especially since fetal development contributes to broad public health outcomes. Many fish species are a rich source of essential nutrients, such as protein, vitamin D, selenium, various minerals, and omega-3 fatty acids [1–4]; the nutritional benefits of fish, including the benefits for fetal cognitive development, are well established [4–6].

Despite evidence of nutritional benefit, American consumers have received seemingly contradictory advice about the nutritional value and methylmercury (MeHg) content of many fish species [7–10]. Furthermore, dietary decisions are also affected by a highly diverse market for fish with significant variability in price and geographic access [11–16].

To address the specific concerns, the FDA’s 2014 report dedicated separate analyses to the positive developmental effects of eating various fish and to the independent developmental effects of MeHg exposure, using both analyses to predict net cognitive outcomes for different patterns of fish consumptionInclusive of MeHg exposure, a consistent association was observed between maternal fish consumption and improvements in neurodevelopmental functioning in U.S. children through 9 years of age [17]. Subsequent modeling yielded estimates of the likely change in a child’s IQ due to the amount of a specific fish consumed during gestation. The curves tended to show a steep increase in predicted IQ, from slightly below average to moderately above average, when moving from low to recommended levels of fish consumption. Net cognitive benefits were consistently observed when consumption during pregnancy was more than 12 oz per week. Peak benefits varied for each type of fish but generally peaked at 1 to 3 meals per week, diminishing slightly at higher frequencies of consumption but maintaining overall cognitive benefit [17].

These data suggest that, for most fish species consumed in the US, recommended dietary levels do not cause significant MeHg exposure [17]. No adverse cognitive effects from MeHg are expected for most fish species when consumed at a level of 1 to 3 meals per week. Moreover, even at significantly higher levels of consumption than recommended, the negative effects of MeHg are predicted to be far smaller than the adverse effects of eating too little fish [17]. For example, about 120 light tuna sandwiches would need to be consumed each week before reaching the minimum MeHg exposure for adverse cognitive effects to be expected [17]. Lastly, there is a considerable difference between the recommended rate of fish consumption for maximum benefits during pregnancy (12 oz of various fish per week) reported in the current FDA advisory and the 2010 Dietary Guidelines for Americans and the amount currently eaten by pregnant women in the U.S. (1.89 oz per week) [17, 18].

Ideally, consuming the amount of seafood that offers peak benefit rates would lead to better cognitive development and health for many Americans. However, recent analyses on economic incentives and consumer attitudes have outlined challenges to adherence of dietary guidance, mainly attributed to concerns over cost and safety [11, 13, 20]. Consumers appear to overestimate risks of contaminant exposure and underestimate the risks of nutrient deficiency, an outcome with a greater likelihood of occurring [4, 21, 22]. Consumers of commercial fish have historically misinterpreted fish MeHg advisories and reduced their consumption of all fish as a “precaution” [4, 21, 23, 24]. This tendency has been vividly demonstrated in surveys and focus groups. Consumers surveyed in Belgium, for example, tended to have a higher awareness of contaminants in fish than of nutrients, and pregnant women in Boston showed a similar ignorance of nutritional benefits, while citing mercury contamination as a reason for avoiding fish altogether [3, 9].

**Fig. 1:**
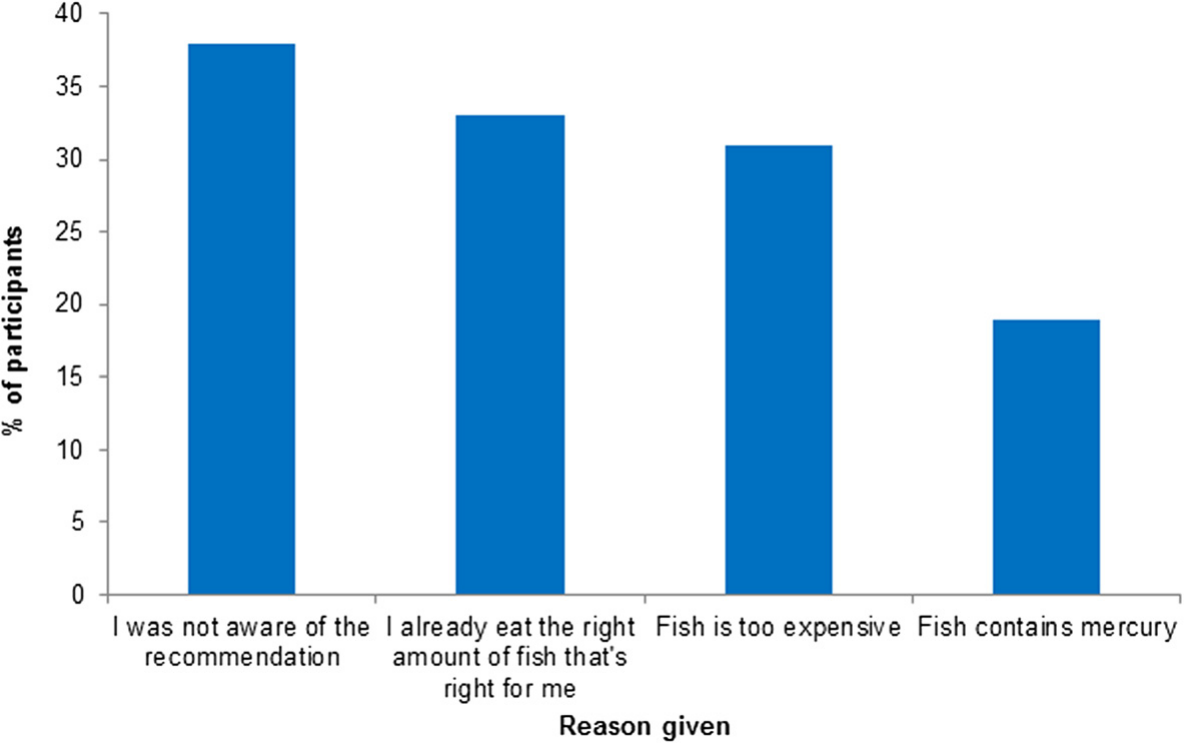
Reasons for not eating the recommended amount of fish (aided), general population survey [25]. Survey participants were asked to check all that apply from four reasons provided to best describe why they do not eat the recommended amount of fish (at least 2 or 3 servings of any variety of fish or seafood each week)

Economic considerations also appear to limit fish intake. For instance, in opinion polling, fish consumption is influenced by the perception that fish are too expensive for routine consumption (Fig. 1) [25]. Though it is inaccurate for many fish species, this perception attests to the real economic forces that drive consumers toward inexpensive fish options.

FDA communications calibrated to emphasize nutritional benefits, appropriately present risk of MeHg exposure, and highlight the affordability of some fish products could have a major social impact and lead to improved dietary practices [4, 21, 26]. To that end, this review will supplement the FDA’s findings with econometric analyses, providing insight into the real-world determinants of dietary decision making and of fish consumption in particular. Below we survey the commercial fish species for which FDA data, pricing data, and regional access data are currently available. Our aim is to inform future advisories and educational initiatives by identifying the products likely to have the greatest value for public health, in terms of their combined nutritional benefits, affordability, and accessibility.

## Materials and methods

Leveraging the FDA’s analysis, we considered only those fish species and market types for which the size of developmental benefits was known, and used the peak increase in childhood IQ by 9 years of age as a proxy for developmental benefits in general. As needed, these data were supplemented with independent studies identified on MEDLINE and PubMed. Of the species for which developmental benefit data was available, a smaller group showed significantly larger peak cognitive benefits and was further described by pricing data (multi-year average costs). A final selection of fish species from this smaller cohort was then directly compared, profiling six commercial fish categories as part of a dietary plan for a pregnant woman and listing cost and consumption rates for maximum benefit and adverse event occurrence. The analysis made no substantive modeling assumptions. In addition, notes on geographic distribution from surveys of food outlets in Orangeburg County, South Carolina, and in the U.S. North Central Region were included to profile regional availability. Orangeburg County was chosen because it covers a large land area containing a varied combination of rural, mixed, and urban Census Tracts. (Liese, 2007) Nielsen Holdings N.V., a global information company that assesses consumer behavior, provided data for multi-year trends for average retail price (ARP), polling on consumer attitudes toward seafood and regional availability. As needed, these data were supplemented by independent studies, identified on WebEc and other online resources.

Fish categories in the pricing data were not always aligned with “species or market type” categories in the FDA report, but in borderline cases a match was assumed. Following convention, we expressed weights of fish in ounces (1 oz = 28.35 g) and defined a fish meal as one serving of fish, which was a minimum of 4 oz. In addition, fish steaks were assumed to weigh 3 to 4 oz.

## Results

Numerous fish species and market types are listed in ascending order of peak IQ benefit (Table 1). The 90 % confidence interval for Gulf tilefish, swordfish, shark, and king mackerel implies that zero consumption of these species may be advisable, but they are exceptions. Recommended consumption (8–12 oz of fish per week as stated in the 2010 Dietary Guidelines and reestablished in the 2015–2020 Dietary Guidelines) showed remarkably little variation across other fish types [24–26, 36]. While peak IQ benefit size ranged from 1.4 to 3.3 IQ points, consumption rate for net IQ detriment varied by nearly two orders of magnitude between the highest (3.3 IQ points) and lowest (1.4 IQ points) values.

**Table 1:** Peak IQ benefits by 9 years of age^a^

Species or market type	Size of maximum benefit expressed as a number of IQ points, Estimate (CI)	Oz. per week to become adverse, Estimate (CI)	Oz. per week to reach maximum benefit, Estimate (CI)
Tilefish, Gulf	1.4 (0.0, 2.6)	16 (0, 30)	8 (0, 13)
Swordfish	2.0 (0.7, 3.0)	24 (12, 43)	8 (7, 13)
Shark	2.0 (0.7, 3.0)	24 (12, 44)	8 (7, 13)
Mackerel, King	2.4 (1.4, 3.2)	32 (16, 59)	8 (7, 13)
Orange Roughy	2.6 (1.7, 3.4)	41 (21, 76)	8 (8, 13)
Grouper	2.7 (1.9, 3.6)	54 (26, 94)	8 (8, 13)
Tuna, Fresh	2.8 (2.1, 3.7)	60 (31, 111)	9 (8, 13)
Mackerel, Spanish	2.8 (2.2, 3.7)	64 (33, 117)	9 (8, 13)
Sable Fish	2.8 (2.2, 3.7)	64 (33, 117)	9 (8, 13)
Bluefish	2.8 (2.2, 3.7)	64 (33, 117)	9 (8, 13)
Tuna, Albacore Canned	2.8 (2.2, 3.7)	67 (35, 123)	9 (8, 13)
Croaker, Pacific	2.9 (2.3, 3.8)	78 (40, 144)	9 (8, 13)
Lingcod & Scorpion Fish	2.9 (2.3, 3.9)	82 (42, 151)	9 (8, 13)
Trout, Saltwater	3.0 (2.3, 3.9)	91 (46, 166)	9 (8, 13)
Bass, Saltwater	3.0 (2.4, 3.9)	95 (49, 174)	9 (8, 13)
Halibut	3.0 (2.4, 3.9)	95 (49, 175)	9 (8, 13)
Carp & Buffalo Fish	3.1 (2.5, 4.0)	139 (71, 254)	9 (8, 13)
Snapper, Porgy & Sheepshead	3.1 (2.5, 4.1)	147 (76, 270)	9 (8, 13)
Perch (ocean), Rockfish, Mullet	3.1 (2.5, 4.1)	157 (81, 288)	9 (8, 13)
Skate	3.1 (2.5, 4.1)	172 (89, 315)	9 (8, 13)
Tuna, Light Canned	3.1 (2.6, 4.1)	296 (101, 360)	9 (8, 13)
Tilefish, Atlantic	3.2 (2.6, 4.1)	214 (110, 392)	9 (8, 13)
Whitefish	3.2 (2.6, 4.1)	235 (121, 432)	9 (8, 13)
Cod	3.2 (2.6, 4.1)	229 (118, 419)	9 (8, 13)
Mackerel, Chub	3.2 (2.6, 4.2)	268 (138, 490)	9 (8, 13)
Croaker, Atlantic	3.2 (2.6, 4.2)	302 (156, 553)	9 (8, 13)
Flatfish & Flounder	3.2 (2.6, 4.2)	310 (160, 568)	9 (8, 13)
Haddock, Hake & Monkfish	3.2 (2.6, 4.2)	351 (181, 644)	9 (8, 14)
Smelt	3.2 (2.6, 4.2)	351 (181, 644)	9 (8, 14)
Crabs	3.2 (2.6, 4.2)	374 (193, 685)	9 (8, 14)
Butterfish	3.2 (2.7, 4.2)	406 (209, 744)	9 (8, 14)
Anchovies, Herring, Shad	3.2 (2.7, 4.2)	471 (243, 863)	9 (8, 14)
Mackerel, Atlantic & Atka	3.2 (2.7, 4.2)	581 (248, 881)	9 (8, 14)
Pollock	3.2 (2.7, 4.2)	636 (328, 1166)	9 (8, 14)
Crawfish	3.2 (2.7, 4.2)	693 (357, 1269)	9 (8, 14)
Trout (freshwater)	3.2 (2.7, 4.2)	736 (379, 1349)	10 (8, 14)
Salmon	3.2 (2.7, 4.2)	1,024 (528, 1876)	10 (8, 14)
Clams	3.2 (2.7, 4.2)	1,024 (528, 1876)	10 (8, 14)
Sardines	3.2 (2.8, 4.3)	1,177 (607, 2158)	10 (8, 14)
Catfish & Pangasius	3.3 (2.7, 4.3)	1,385 (714, 2539)	10 (8, 14)

Abbreviations: *CI* 5 to 95 % confidence interval, *IQ* intelligent quotient, *oz.* ounces

^a^Excerpted from 2014 FDA Report (Table V-7)

Two features of Table 1 warrant special mention. First, when the four fish with the highest mean concentration of MeHg—gulf tilefish, swordfish, shark, and king mackerel—are excluded, the “best” and “worst” fish types differ in peak benefit by only 0.7 IQ points (2.6 to 3.3 IQ points), and therefore offer comparable developmental benefits. Second, the species with the highest mean concentration of MeHg (gulf tilefish), would need to be eaten at twice the rate predicted to achieve peak IQ benefit in order for adverse effects to be seen. For each other fish type, rates of consumption to experience adverse effects are more than three times greater than the rate needed to reach peak IQ benefit.

To assess the cost of consuming enough fish to achieve an IQ benefit, we reported the mean average cost of each fish variety per pound (ARP) converted to price per ounce over 2009–2013 or 2010–2013 (Table 2). Canned salmon and tuna were the least expensive with average cost per ounce of $0.20 and $0.26, respectively. All listed tuna products provide peak benefits between 2.8 and 3.1 IQ points, while maternal consumption of salmon provides 3.2 IQ points to a child.

**Table 2:** Multi-year average costs^a^

Species or market type	Average cost/oz., fresh	Average cost/oz., canned
Anchovy, Herring, Shad	--	$0.65
Bass, Saltwater	$1.11	--
Catfish, Pangasius	$0.26	--
Cod	$0.39	--
Flatfish, Flounder	$0.31	--
Haddock, Hake, Monkfish	$0.44	--
Halibut	$1.01	--
Perch, Ocean, Rockfish, Mullet	$0.33	--
Salmon	$0.45	$0.26
Sardines	--	$0.27
Snapper, Porgy, Sheepshead	$0.47	--
Trout, Freshwater	$0.42	$0.83
Trout, Saltwater	$0.42	$0.83
Tuna, Albacore	--	$0.30
Tuna, Fresh	$0.49	--
Tuna, Light	--	$0.20

^a^Excerpted from Nielsen 2014 [25]

We then calculated the number of meals per week and attendant costs of select fish species necessary to attain maximum nutritional benefit for a representative pregnant woman. Canned light tuna demonstrated the lowest cost per week for maximum benefit ($1.83), followed by canned salmon ($2.61) and canned albacore tuna ($2.72) (Table 3). These individual meal-per-week rates for maximum benefit are currently followed by only 5 to 25 % of U.S. men and women [17].

**Table 3:** Dietary plans for a pregnant woman

Species or market type	Meals per week for maximum benefit	Meals per week for possible adverse effects	Cost per week for maximum benefit	Cost per week as a multiple of cost for canned light tuna
Anchovies, Herring, Shad	3	~150	$5.82	3.18
Fresh Salmon	3–4	~330	$4.52	2.47
Fresh Cod	2–3	~70	$3.50	1.91
Canned Salmon	3–4	~330	$2.61	1.43
Canned Albacore Tuna	2–3	~20	$2.72	1.49
Canned Light Tuna	2–3	~90	$1.83	1

Finally, to briefly assess fish availability at food outlets in several U.S. regions, we report the availability of fresh and canned or pouched fish in common food outlets. Findings reveal that canned seafood is widely available in supermarkets, grocery stores, and convenience stores and found at a greater percentage at these locations than fresh seafood. Its data support the larger market presence of canned fish compared with fresh fish in more remote and rural areas especially.

## Discussion

The results of this preliminary analysis indicated that canned and pouched fish products are low-cost options for consumers that can provide substantial cognitive benefits to the developing fetus. Because of the relatively low cost and wide distribution, canned tuna and other canned fish products could be eaten with regularity to provide peak cognitive benefits. By contrast, a large proportion of commercial fish are substantially more expensive, hindering consumer efforts to follow dietary guidance [12, 14–17]. Here we discuss each of these important sub-results, assessing validity and significance through relevant literature.

## Developmental benefit

The FDA’s peak cognitive benefit data list several fish species that, when part of a pregnant woman’s diet, offer average childhood IQ increases of up to 3.3 points. This is a substantial increase, and all the more encouraging given that several of the most beneficial fish species are some of the most consumed [16, 17]. For example, fresh tuna was associated with a benefit of 2.8 IQ points at recommended consumption levels, while light canned tuna offers 3.1 points, the same value as for carp, skate, snapper, and perch (Table 3).

**Table 4:** Notes on geographic distribution

Market type	Relevant literature
Fresh Fish	In a survey of food outlets in Orangeburg County (OC), South Carolina, fresh seafood was available at 82, 63, and 0 % of supermarkets, grocery stores, and convenience stores, respectively [14].
	In a separate survey of food outlets in the U.S. North Central Region (NCR), 28 % of supermarket seafood sales were fresh products [15].
Canned Fish	Canned seafood was available at 100 %, 100 %, and ≥54 % of OC supermarkets, grocery stores, and convenience stores, respectively [14].
	17 % of NCR supermarket sales were pre-packaged/branded^a^ products. 26 % of all surveyed NCR rural supermarkets sold *only* pre-packaged/branded seafood [15].
Canned Salmon	Canned salmon in water was available at 100, 100, and 23 % of OC supermarkets, grocery stores, and convenience stores, respectively [14].
Canned Tuna	Canned tuna in water was available at 100, 100, and 54 % of OC supermarkets, grocery stores, and convenience stores, respectively [14].

^a^ Branded seafood products refer to pre-packaged items from companies such as Gorton’s^TM^ or Mrs. Paul’s^TM^

Such results are consistent with the well-characterized nutrition benefits of fish in general. There is a robust evidence base supporting the independent benefits of omega-3 fatty acids, which are essential structural components of neurons, among other cell types, and can only be obtained through dietary sources, including many commercial fish species^1^. Indeed, the FDA report separately considered the possibility that nutrition benefits for each fish type are due chiefly to omega-3 fatty acids and offered a preliminary analysis of developmental effects due to omega-3 fatty acids alone [17]. When omega-3 fatty acids are the sole source of the beneficial effect from fish consumption, a much larger serving size per week is needed to reach peak IQ benefit for most species [17]. Current literature also links omega-3 fatty acids and omega-3 fatty acid subtypes with various postnatal benefits, including longer gestation and increased birth weight; however, distinctions between dietary fish and fish oil supplements have yet to be confirmed [27]. Other nutrients found in fish such as vitamin D, selenium, and the essential amino acids found in protein offer developmental benefits that cannot be achieved with fish oil supplementation exclusively [28].

Regardless of the biochemical basis for the observed population-level cognitive benefits, the results complement an emerging consensus that early neurological development can be enhanced by dietary fish and that this enhancement probably reflects the full nutritional profile of fish [2, 29]. Indeed, a suite of studies have also linked greater maternal fish intake with improved neurological development on various measures. In a seminal study by HibbeIn et al., fish intake below 12 oz per week in pregnant women was associated with an elevated risk of having children in the lowest quartile of verbal development; similar results were obtained for prosocial behavior, fine motor skills, communication, and social development [19]. Independent studies have characterized a similar relationship for closely related measures of motor skills and vocabulary, and found marked benefits in verbal development at 15 months for infants of mothers who ate more than four fish meals per week [30, 31].

## Cost and accessibility

The FDA analysis has quantified the developmental benefits of fish consumption, but economic factors are also key determinants of consumer choice. With this in mind, our results include economic data and an assessment of regional availability, which identify the fish species most likely to deliver population-wide cognitive benefits. The objective was to assess not just the nutrient value, but also the convenience of the food source.

Our analysis focused on cost and accessibility, which are both widely accepted determinants of food convenience [20]. The results presented here demonstrate that both cost and accessibility vary greatly among fish that provide strong cognitive benefits. The monthly cost of providing peak developmental benefits for a given commercial fish varied by a factor of more than 3. The substitution of canned tuna for fresh salmon, as a source of peak cognitive benefits, was shown to save an average adult nearly $3 per week in discretionary food spending—a figure whose social implications will be discussed below. Benchmarks for cost are readily available and can highlight its practical meaning for consumers. For example, the difference in cost between canned tuna and fresh salmon at recommended rates of consumption, is $130 annually, or nearly 6 % on average for the typical adult [32]. Similarly, recommended annual rates of canned tuna provide 6.5 g of protein per ounce, similar to the protein content of beef (6.0), less than chicken (8.3), and more than pork (3.5) [32]. This helps to make tuna not only metabolically, but financially practical as a dietary source of protein; a gram of protein from canned tuna costs roughly the same as a gram from beef and considerably less than a gram from pork [33]. In fact, roughly $100 per year, or 4.5 % of at-home food expenses for an average adult would be saved by substituting pork with canned tuna at recommended levels of consumption [33]. The savings are even more dramatic when the number of consumers per household is increased and the household income is decreased. Substituting canned tuna for fresh salmon based on cognitive benefits would save a family of four roughly 7.6 % of its annual food budget per year, while a substitution of canned tuna for pork based on protein content would save roughly 5.8 % per year.

The availability of fish products at food outlets showed similar disparities, particularly in rural areas. In a survey of food outlets in Orangeburg County, South Carolina, canned seafood was available in 100 % of supermarkets and grocery stores surveyed and in more stores than fresh seafood [14]. In a separate survey of food outlets in the U.S. North Central Region, 17 % of supermarket sales were pre-packaged/branded products, and 26 % of all surveyed rural supermarkets sold only pre-packaged/branded seafood [15].

Cost and availability does not, of course, preordain consumers’ final selection of individual products. Other factors—including taste preferences and ease of cooking—also influence everyday dietary decision making. Still, the influence of both cost and local availability on the selection of food products generally, and of fish in particular, is thought to be strong. Prohibitive costs and sparse distribution present especially formidable barriers to fish consumption for low-income and rural consumers [11, 12, 14, 34].

These and other economic analyses underscore the potential benefits of public health advice and educational programs developed to educate consumers on optimal fish consumption. The results reported here suggest that fish products, specifically canned light tuna, with substantial nutritional benefits can be realistically adopted as dietary staples by most consumers. Addressing cost, availability, and other practical concerns could allow advisories to provide a broader heuristic framework to encourage consumers to increase their fish intake and meet the 12-ounce-per-week recommendation [18].

## Behavioral rationale

Such recommendations could also serve to balance the consumer’s tendency to use maladaptive shortcuts and overlook the context for dietary advice, such as avoiding foods with traces of contaminants to the detriment of overall health [4, 22, 23, 35]. This tendency is manifest in the bias against many fish products [4, 21, 23, 24]. In general, consumers deviate from health-optimal behavior because of the perception that there is a tradeoff between access and quality.

Environmental concerns may also contribute to lack of confidence and clarity surrounding fish consumption. Some advocacy groups have recommended avoiding consumption of certain types of fish, citing concerns about species depletion or habitat destruction. A focused perspective on reducing ecological harm can lead to contradictory advice; for example, farm-raised salmon in the diet is encouraged for its high omega-3 and low MeHg content, however some environmental groups suggest eliminating its consumption because of concern over how salmon aquaculture can affect the marine ecosystem [4]. The 2015 Dietary Guidelines for Americans Advisory Committee Report considered both the nutritional and sustainability aspects of seafood and concluded that farm-raised and wild versions of the same species are generally nutritionally similar, safe, and complement each other to meet demand for seafood now and moving forward. The Advisory Committee claim relies on the continuation of a projected 33 % increase in global aquaculture output by 2021, a rate which would raise the total supply of seafood by 15 % [36]. This increased supply would allow for global consumption to meet Dietary Guideline recommendations for consumption (at least 8 oz of seafood per week), if distributed evenly to the world population.

We submit that a strong commitment to effective messaging, with emphasis on the nutritional value of affordable fish products, can equip consumers with the necessary information to make health-optimal choices about the fish they eat. There are ample grounds for optimism about this approach; many pregnant women in focus groups reported hearing no advice to eat fish while pregnant, and broader consumer surveys have revealed that the desire for health benefits is one of the main drivers of consumption behavior [3, 9, 13]. Low-income and rural consumers might stand especially to benefit, since their dietary options for fish are often limited to the fish species whose health benefits are rarely publicized [4, 14, 21]. An integrated health outreach strategy that incorporates the FDA’s findings within their broader societal context would include advice stressing the nutritional benefits of affordable and accessible fish products and limiting the risk of developmental impairment from a low-fish diet or from harmful exposure to MeHg.

These findings help illustrate the benefits of a consumer-centric analysis, but are not without limitations. Their cost projections are simple averages over 4 to 5 years of recent data; sources of volatility in future pricing were beyond the scope of this analysis. Geographic resolution was also limited, relying on a handful of regional surveys to represent national trends in fish pricing and distribution (Table 4).

## Conclusions

American consumers urgently need educational messages and materials that stress the healthful benefits of affordable and convenient fish. The under-consumption of fish, due partly to misconceptions about contaminant levels and due partly to perceived affordability barriers, can adversely affect the health and development of millions. An evidence-based approach to these unmet health needs should address the unintended consequences of the past and the decision making of real consumers. It would highlight the benefits of canned and pouched fish products, including canned and pouched tuna products, since, on several key indices, these products are most likely to deliver the developmental benefits that have so far eluded many Americans.

These products were found to offer high developmental and health benefits, while appearing in more food outlets for lower prices, ultimately saving individual consumers up to $130 per year in discretionary food spending. Such savings could improve the economic bottom line for low-income consumers and influence the dietary choices of nearly all consumers. Though the effect on individual purchasing choices may be subtle, these price differences may cumulatively favor the incorporation of fish products into a regular diet, ideally at levels where their health benefits can be fully realized.

Supplementing current nutrition data with research on consumer preferences and behavior is a likely first step in developing a new program for dietary guidance. A new dietary program rooted in empirical, market-based research could optimize health outcomes nationwide. These results suggest that a thorough consideration of market-based factors can provide dietary guidance based on the everyday considerations that influence consumer choice. In particular, they would likely reveal the need for a realignment of dietary guidance with a consumer-centric assessment of the risks and the benefits of fish consumption.

## Abbreviations

ARP, average retail price; FDA, Food and Drug Administration; MeHg, methylmercury

## Footnotes

1 Beyond development, the role of omega-3 fatty acids in maintaining cardiovascular health is well-documented. (13)
